# Supplementary material for: Association of Thermoresponsive Diblock Copolymer PDEGMA-b-PDIPAEMA in Aqueous Solutions: The Influence of Terminal Groups
Source: Polymers (Basel). 2024 Jul 24;16(15):2102. doi: 10.3390/polym16152102 (PMC11313919; doi:10.3390/polym16152102)
Supplement: Supplementary file 1 [file polymers-16-02102-s001.zip › polymers-3081350-supplementary.pdf]

Supporting Information

Association of thermoresponsive diblock copolymer PDEGMA-PDIPAEMA in aqueous solutions: The influence of terminal groups

Adam Škorňa[1], Dimitrios Selianitis[2], Stergios Pispas[2] and Miroslav Štěpánek[1]

[1]*Department of Physical and Macromolecular Chemistry, Faculty of Science, Charles University, Hlavova 2030, 128 40 Prague 2, Czech Republic*

[2]*Theoretical & Physical Chemistry Institute, National Hellenic Research Foundation, 48 Vassileos Constantinou Avenue, 11635 Athens, Greece*

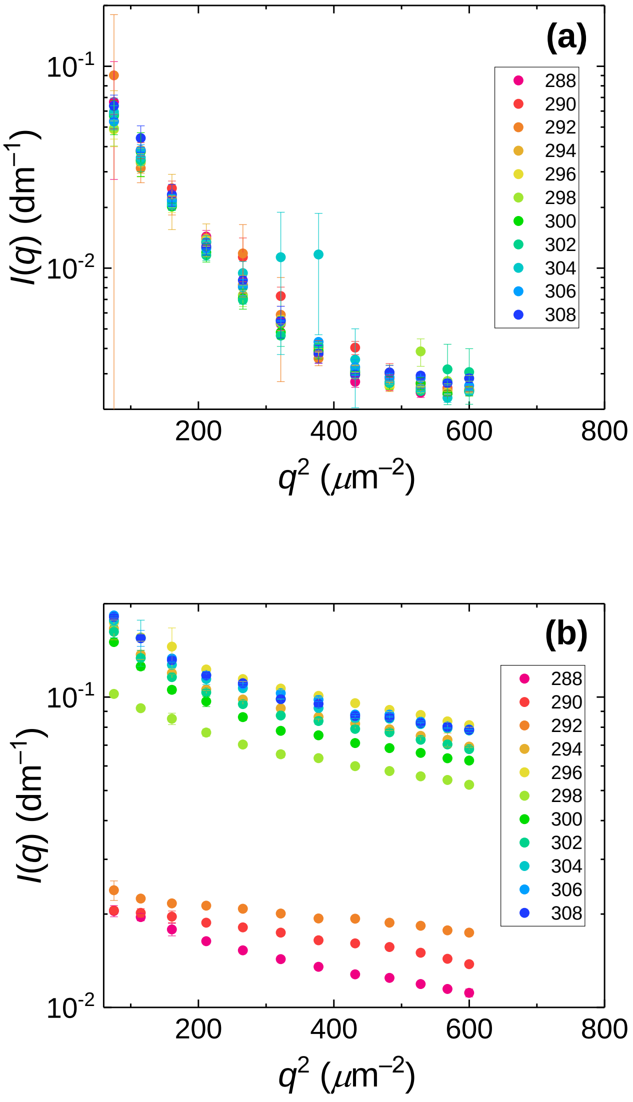

Figure S1. Guinier plots of static LS of PDEGMA-PDIPAEMA solutions at (a) pH=2 and (b) pH=7.

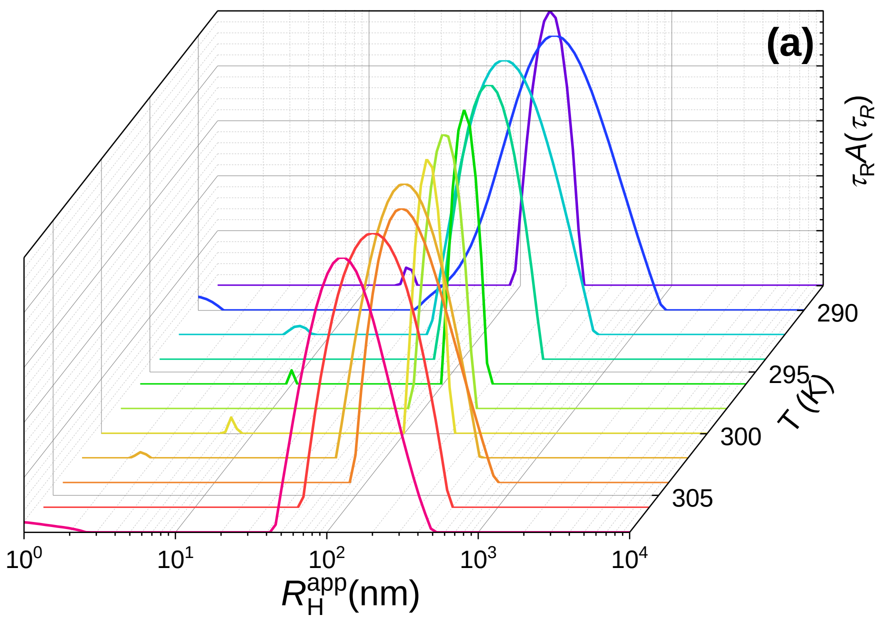

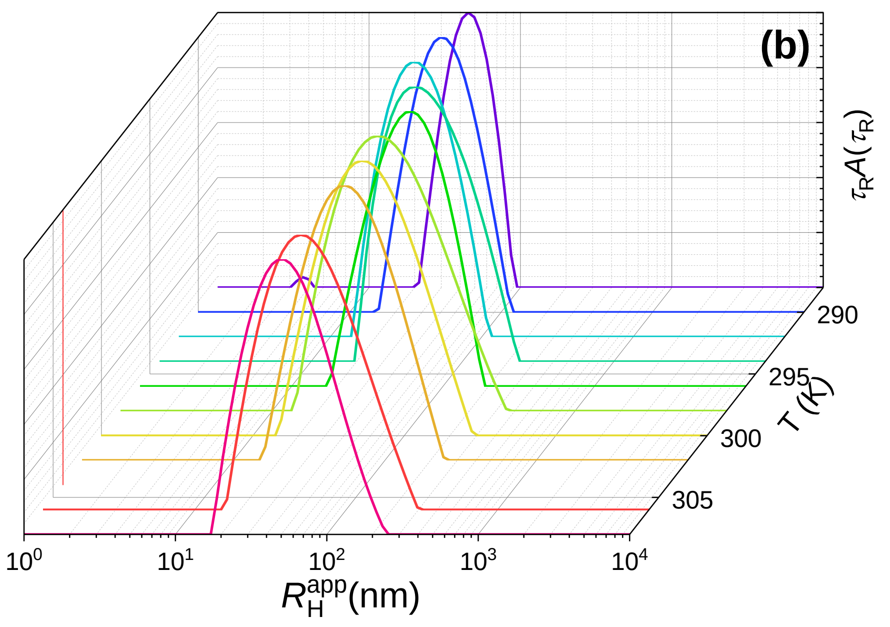

Figure S2. CONTIN distributions of hydrodynamic radii obtained from dynamic LS of PDEGMA-PDIPAEMA solutions at pH=2 and pH=7.
